# Interactions between Exosomes from Breast Cancer Cells and Primary Mammary Epithelial Cells Leads to Generation of Reactive Oxygen Species Which Induce DNA Damage Response, Stabilization of p53 and Autophagy in Epithelial Cells

**DOI:** 10.1371/journal.pone.0097580

**Published:** 2014-05-15

**Authors:** Sujoy Dutta, Case Warshall, Chirosree Bandyopadhyay, Dipanjan Dutta, Bala Chandran

**Affiliations:** H. M. Bligh Cancer Research Laboratories, Department of Microbiology and Immunology, Chicago Medical School, Rosalind Franklin University of Medicine and Science, North Chicago, Illinois, United States of America; Meharry Medical College, United States of America

## Abstract

Exosomes are nanovesicles originating from multivesicular bodies and are released by all cell types. They contain proteins, lipids, microRNAs, mRNAs and DNA fragments, which act as mediators of intercellular communications by inducing phenotypic changes in recipient cells. Tumor-derived exosomes have been shown to play critical roles in different stages of tumor development and metastasis of almost all types of cancer. One of the ways by which exosomes affect tumorigenesis is to manipulate the tumor microenvironments to create tumor permissive “niches”. Whether breast cancer cell secreted exosomes manipulate epithelial cells of the mammary duct to facilitate tumor development is not known. To address whether and how breast cancer cell secreted exosomes manipulate ductal epithelial cells we studied the interactions between exosomes isolated from conditioned media of 3 different breast cancer cell lines (MDA-MB-231, T47DA18 and MCF7), representing three different types of breast carcinomas, and normal human primary mammary epithelial cells (HMECs). Our studies show that exosomes released by breast cancer cell lines are taken up by HMECs, resulting in the induction of reactive oxygen species (ROS) and autophagy. Inhibition of ROS by N-acetyl-L-cysteine (NAC) led to abrogation of autophagy. HMEC-exosome interactions also induced the phosphorylation of ATM, H2AX and Chk1 indicating the induction of DNA damage repair (DDR) responses. Under these conditions, phosphorylation of p53 at serine 15 was also observed. Both DDR responses and phosphorylation of p53 induced by HMEC-exosome interactions were also inhibited by NAC. Furthermore, exosome induced autophagic HMECs were found to release breast cancer cell growth promoting factors. Taken together, our results suggest novel mechanisms by which breast cancer cell secreted exosomes manipulate HMECs to create a tumor permissive microenvironment.

## Introduction

Breast cancer is a leading cause of cancer death in women worldwide. Approximately, 1 out of every 8 women is expected to be diagnosed with breast cancer in their lifetime [1]. In spite of great strides made in diagnosis for breast cancer in the last decade, treatment options remain limited particularly since little is known about how primary breast tumors develop in the mammary ducts and how the primary tumor subsequently progresses as an invasive and metastatic disease [2], [3]. Recent data suggests that the tumor microenvironment (TME) plays a critical role in disease initiation and its progress [4]–[8]. The TME is composed of several cell types depending on the stage of tumor development. During the initial stages of tumor development and in the case of tumors *in situ*, TME is largely composed of ductal epithelial and myoepithelial cells, while in the later stages of tumor progression, namely, during invasive disease, TME is composed of several cell-types such as fibroblasts, endothelial cells, mammary epithelial cells, adipocytes and immune cells [5],[7],[9]. Several recent studies have indicated that cancer associated fibroblasts and immune cells present in the TME communicate with breast cancer cells to facilitate tumorigenesis [6], [10]–[13]. While it has been shown that the nature of intercellular communications or “cross talk” between TME and breast cancer cells affects how the tumor responds to anti-cancer therapeutics [14]–[16], precise mechanisms of intercellular communications are still not clearly understood. More specifically, completely unknown is the “cross talk” mechanism between the breast cancer cells and the normal epithelial cells of the mammary duct during tumor development and progression.

Cancer cells and TME are known to communicate with each other not only via direct contact (by adhesion factors) but also by secreted paracrine factors (released factors) such as secreted proteins (cytokines and pro-angiogenic factors), nucleic acids and extracellular vesicles (EVs) [8],[17],[18]. Among the released factors, EVs represent a new paradigm of intercellular communications [18]. EVs have a size range of 50 to 1000 nm and are further categorized into microvesicles/apoptotic bodies, membrane particles, exosome like vesicles and exosomes based on their size, origin and molecular composition [19]. Exosomes are multivesicular body-derived vesicles of 50 to100 nm in diameter and were first described as such by Johnstone et al., in 1987 [20]. These vesicles contain a wide range of functional proteins, mRNAs and miRNAs and are actively secreted via exocytosis from almost all cell types including dendritic cells, lymphocytes, and tumor cells [21], [22].

Exosomes are found in almost all physiological fluids including urine, plasma, saliva, semen and breast milk since their small size allows them to travel freely across tissue spaces and in the circulatory system [23]–[25]. Furthermore, since exosomes bear the molecular signatures of the cell of origin, they have been widely studied for the development of biomarkers [26], [27]. However, several recent studies have demonstrated that exosomes may act as mediators of intercellular communications affecting various physiological and pathophysiological processes [28]–[30]. Intercellular communications mediated by exosomes are primarily achieved via either one or multiple mechanisms of exosome-target cell interactions. Exosomes have been shown to interact with target cells by specific receptor-ligand engagements sometimes leading to their uptake by target cells while simultaneously triggering specific intracellular signal cascades (i.e., via juxtacrine signaling), which often leads to alterations of gene expression in these target cells [31], [32]. Other mechanisms of exosome-target cell interactions include their uptake either by phagocytosis or fusion of exosomal membranes with target cell plasma membranes [33]–[35]. Regardless of the involved mechanisms, exosomal cargo has been shown to be delivered into cytosolic compartments and often also ends up in the nuclei of target cells [36].

In the context of tumor development, exosome-mediated signaling has been shown to promote tumor progression through communications between the tumor and its surrounding stroma [37], activation of proliferative and angiogenic pathways [38], initiation of premetastatic sites [39], [40], and suppression of the immune-surveillance machinery [41]. In breast cancers, tumor secreted exosomes have been shown to facilitate tumor progression and metastasis by affecting cancer cell adhesion and spreading [42], transfer of phenotypic traits to secondary cells [43], converting adipose tissue derived mesenchymal stem cells into myofibroblast like cells [44], and by inhibiting differentiation of bone marrow dendritic cells [45]. In addition to tumor secreted exosomes, those secreted by normal cells of the TME have also been shown to facilitate tumor development and metastasis by acting upon the breast cancer cells [46], [47]. However, completely unknown are the effects of breast cancer cell secreted exosomes on the normal mammary epithelial cells which are one of the key members of the ductal microenvironment and are also found in TME of invasive disease.

In this study, we determined how breast cancer cell released exosomes manipulate human primary mammary epithelial cells (HMECs) to facilitate tumor growth. We show that exosomes released from breast cancer cells are taken up by HMECs and exosome-HMEC interactions results in ROS production. ROS induces autophagy, DNA damage response (DDR), phosphorylation of p53 at serine 15 and stabilization of p53 in HMECs. Treatment of HMECs with the ROS inhibitor N-acetyl-L-cysteine (NAC) not only abrogates ROS production during exosome-HMEC interaction, but also abrogates autophagy, DDR and phosphorylation of p53. Functionally, we show that spent culture media from exosome induced autophagic HMECs can stimulate growth of different breast cancer cell lines, indicating the release of tumor promoting factors by autophagic HMECs.

## Materials and Methods

### Cells

Breast cancer cell lines MDA-MB-231 [48] and MCF7 [49] were cultured in RPMI1640 medium (Life Technologies, Carlsbad, CA) supplemented with 10% FBS and MEM non-essential amino acids (Life Technologies). T47DA18 breast cancer cells [50], were cultured in RPMI1640 medium supplemented with 10% heat inactivated fetal bovine serum (FBS), MEM non-essential amino acids and recombinant human insulin (Life Technologies). Media used for all cell lines were supplemented with 2 mM L-glutamine and antibiotics (penicillin and streptomycin). Normal human primary mammary epithelial cells (HMEC) derived from normal adult mammary glands (Cell Applications, San Diego, CA) were grown in epithelial cell culture media with growth factors (Cell Applications) HMECs were obtained as passage 5, cells between passage 6–9 were used in this study. All cultures were maintained at 37°C in a 5% CO_2_ incubator.

### Antibodies and reagents

Rabbit polyclonal antibodies against human LC3 A/B were from Serotech, Hercules, CA. Rabbit antibodies against human phospho-p53 (S9, S15, S46 and S392, respectively), p53, phospho-histone H2AX (γH2AX) (Serine 139), H2AX phospho-ATM (S1981), ATM, phospho-Chk1 (S345), Chk1, and mouse monoclonal antibodies against human Alix were from Cell Signaling Technologies, Beverly, MA. Mouse monoclonal antibodies against human phospho-histone H2AX (Serine 139) for immunofluorescence assay (IFA) was from Chemicon/Millipore Billerica, MA. Rabbit and mouse anti-human Tsg101 antibodies were from Abcam, Cambridge, MA. Mouse anti-actin and anti-tubulin antibodies were from Sigma, St. Louis, MO. Anti-mouse and anti-rabbit secondary antibodies linked to HRP, Alexa Flour 488, Alexa Flour 350, Alexa Flour 594, Alexa Flour 647 and SlowFade (with or without DAPI) were from Life Technologies. Anti-mouse and anti-rabbit secondary antibodies linked to IRDye 700 or IRDye 800 were from LI-COR Biosciences, Lincoln, NE. N-acetyl-L-cysteine (NAC) was from Sigma.

### Exosome isolation

Exosomes were isolated from cell conditioned media by ultracentrifugation (Fig. S1) [51]. Briefly, cells grown in complete media were trypsinized, washed in PBS extensively and seeded in exosome depleted cell culture media for collecting exosomes. Complete cell culture media containing 20% FBS was centrifuged for 16 h at 100,000×g, supernatants were filtered through a 0.22 µm sterile filter and subsequently mixed with serum free media to prepare exosome depleted cell culture media containing 10% FBS. Cells were grown in exosome depleted culture media up to 70% confluence. Cell culture media was collected and cleared of debris and non-exosome vesicles by sequential centrifugations (200×g for 10 min, 2000×g for 20 min, 10000×g for 30 min) and then centrifuged at 100,000×g for 70 min to pellet exosomes. The exosome pellet was washed 2 times with calcium and magnesium free phosphate buffered saline (PBS) by centrifugation at 100,000×g for 70 min and resuspended in 200 µl PBS.

To study uptake of breast cancer cell released exosomes by normal primary cells, exosomes were labeled with fluorescent dye PKH-67 using the PKH-67 labeling kit (Sigma) [52]. Briefly, 100 µg protein equivalents of exosomes were resuspended in 100 µl PBS and mixed with 100 µl of PHK67 dye diluted in diluent C (1∶1 v/v) for 5 min. This mixture was diluted with 4.5 ml of PBS and centrifuged at 100,000×g for 70 min to pellet the PKH-67 labeled exosomes. The exosome pellet was further washed twice with PBS by ultracentrifugation at 100,000×g for 70 min, to remove any free dye and finally the exosome pellet was resuspended in 100 µl PBS and used for uptake studies.

### Electron microscopy

Exosomes were analyzed by transmission electron microscopy using negative staining. 2.5 µl of purified exosomes was adsorbed onto Formvar/carbon coated copper mesh grids, washed with PBS, and stained with freshly prepared 2.0% phosphotungstic acid in aqueous suspension. Samples were imaged using a JEM-1230 transmission electron microscope (JEOL, Japan) equipped with a LaB6 cathode and operated at an acceleration voltage of 80 kV. Images were taken using a Hamamatsu ORCA- HR CCD (AMT, Massachusetts, US).

### Flow cytometry

Aliquots of 10^5^ target cells in 500 µl serum free media were incubated with 10 µg PKH-67 labeled exosomes for varying time periods at 37°C, washed twice in ice cold PBS, trypsinized, washed by centrifugation at 250×g for 5 min and analyzed by a flow cytometer (LSRII, BD biosciences) using FACS DIVA and Flow Jo software for the uptake of exosomes.

### Immunofluorescence microscopy (IFA)

Cells were grown to semi-confluence in 8- well chamber slides and incubated with exosomes for up to 24 h. Cells were washed extensively with PBS, then fixed and permeabilized using Cytoperm-Cytofix (BD Biosciences) at 4°C for 30 min. For immunostaining, cells were washed using Permwash buffer (BD Biosciences), blocked using 5% normal donkey serum or 5% BSA and sequentially incubated for 1 hr at room temperature, first with primary antibodies and then with secondary antibodies in Permwash buffer. Nuclei were stained with 4′,6-diamidino-2-phenylindole (DAPI) and slides were viewed under epifluorescence microscope (Nikon 80i). Images were captured using a CCD camera and analyzed using Metamorph software.

### Effects of exosome-HMEC interactions

10^5^ HMECs were seeded per well of 6 well plates in epithelial cell complete media for 16 h. Semi-confluent layers of cells were then washed extensively with PBS and epithelial cell basal media (without growth factors) was added to the wells and cells were incubated at 37°C under 5% CO2 for 2 h. After incubation, the media was withdrawn and replaced with epithelial cell basal media either containing 10 µg protein equivalents of exosomes per ml, or without it. Cells were further incubated for up to 24 h and processed for further studies as required.

### Breast cancer cell culture in conditioned media from exosome treated HMECs

Spent culture media from HMECs treated with exosomes for 24 h or untreated, as described in the preceding section was collected, passed through a 0.22 µm filter and used for culturing breast cancer cells. Cancer cells grown in complete media were trypsinized, washed extensively with PBS and seeded in conditioned media from HMEC cultures. Cell density was calculated 24 h later following trypsinization and counting of cells using a haemocytometer.

### Western blot

Cells and exosomes were lysed in non-denaturing cell lysis buffer (20 mM Tris HCL pH 8, 137 mM NaCl, 10% glycerol, 1% NP-40, 2 mM EDTA, protease and phosphatase inhibitors) followed by sonication on ice. Lysates were centrifuged at 14,000×g, for 30 min at 4°C and supernatants were resolved by SDS-PAGE, transferred to nitrocellulose membranes, blocked with 5% skim milk and immunoblotted with the indicated antibodies. Species specific secondary antibodies conjugated to horseradish peroxidase (HRP), IRDye 700 or IRDye 800 were used for detection. Immunoreactive bands detected using HRP conjugated secondary antibodies were visualized using enhanced Chemiluminescent substrate (Pierce, Rockford, IL). Bands were further scanned and quantitated using the Alpha-Imager (Alpha Innotech Corporation, San Leonardo, CA) imaging system. Bands detected using IRDye conjugated antibodies were visualized and analyzed using an Odyssey scanner from LI-COR. Protein estimation in lysates was carried out using the BCA protein assay kit, Pierce.

### ROS measurement

HMECs were cultured in a 96-well plate until they were semi-confluent (70% confluent) and were incubated with epithelial cell basal media without growth factors for 2 h. Cells were loaded with dye by replacing the basal medium with fresh basal media containing 10 µM cell permeant 5-(and-6)-chloromethyl-2′,7′-dichlorodihydrofluorescein diacetate, acetyl ester (CM-H2DCFDA [C6827]; Life Technologies) and with or without 10 µg/ml exosomes for up to 3 h at 37°C under 5% CO2. Fluorescence was measured using a Synergy HT microplate reader (BioTek Instruments, Winooski, VT) with a 485/20 excitation, 528/20 emission filter pair and a photomultiplier tube (PMT) sensitivity setting of 50. Between each two time points, the cells were kept in the culture incubator. For measurement of ROS in the presence of NAC, cells were treated with 1 µM NAC for 1 hr in epithelial cell basal media, washed and incubated with exosomes in the presence of NAC and ROS detector CMH2DCFDA and processed as described above.

### Statistical analysis

All experiments were carried out in triplicate and repeated at least twice. Histograms represent the mean values, and bars indicate standard error of the mean. The statistical significance of the results was determined using Student's t-test and Anova. The data was considered significant when *p*<0.05.

## Results

### Exosomes secreted by breast cancer cells are taken up by normal human primary mammary epithelial cells (HMECs)

To study the effects of breast cancer cell secreted exosomes on normal mammary epithelial cells, we isolated exosomes from conditioned media of 3 different breast cancer cell lines (MDA-MB-231, T47DA18 and MCF7) using multi-step centrifugation (Fig. S1) [51]. These cell lines were chosen to represent three different types of breast cancers, viz., MDA-MB-231, triple negative and highly aggressive metastatic adenocarcinoma [48]; T47DA18, estrogen receptor positive and invasive ductal carcinoma [50]; MCF7, estrogen receptor positive but non-aggressive metastatic adenocarcinoma [49]. Exosome yields from multiple independent batches of cell cultures were estimated by assessing the total protein content using BCA methods.

**Figure 1:**
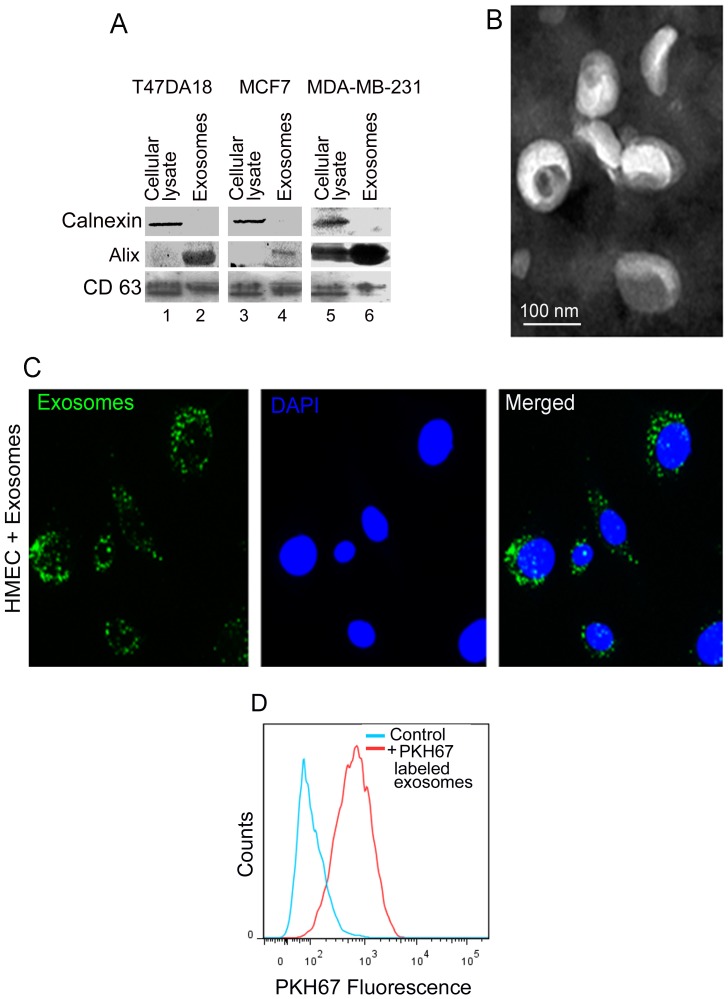
Characterization of exosomes secreted by breast cancer cells and exosome uptake by HMECs. Exosomes were isolated from conditioned media of 3 different breast cancer cell lines, T47DA18, MCF7 and MDA-MB-231 and characterized by (A) detection of exosome specific proteins by western blotting and (B) electron microscopy. (A) Western blotting for endoplasmic reticulum specific protein calnexin and exosome marker proteins Alix and CD63 in total cellular lysates (lanes 1, 3 and 5) and exosome preparations (2, 4 and 6). 10 µg of protein was analyzed for each sample. (B) Characterization of exosomes from MDA-MB-231 cells by transmission electron microscopy (TEM). Exosomes isolated by multi-step centrifugation were fixed, negatively stained using phosphotungstic acid and observed by TEM. (C) Exosomes isolated from conditioned media of MDA-MB-231 cells were labeled with fluorescent dye PHK67. 10 µg protein equivalent of labeled exosomes were incubated with 5×10^4^ HMECs for 24 h. HMECs were washed extensively with PBS, fixed in PFA and observed using an epifluorescence microscope. Green fluorescent “specs” represent PKH-67 labeled exosomes taken up by the HMECs. (D) Flow cytometric analysis of HMECs exposed to PKH-67 labeled exosomes as described in (C).

In our experiments, for all 3 different cancer cell lines, we routinely isolated approximately 200 µg protein equivalent of exosomes from 240 ml of conditioned media collected from cultures of 45×10^6^ cells. We assessed the purity of isolated exosomes using western blotting to detect the presence of well known exosome marker proteins, Alix [51] and CD63 [51] as well as the absence of calnexin, an endoplasmic reticulum resident protein that is often associated with cell debris [51]. As expected, calnexin was only detected in total cellular lysates and not in exosomes (Fig. 1 A, lanes 1, 3 and 5 vs. lanes 2, 4 and 6), indicating that our exosome preparations are free of cellular components and debris. Furthermore, both Alix and CD63 were detected in exosomes derived from all 3 different cancer cell lines (Fig. 1 A, lanes 2, 4 and 6). We also observed that while CD63 was present in both cellular lysates and exosomes for all the cell types, Alix was not significantly detectable in lysates of T47DA18 and MCF7 cells when compared to the MDA-MB-231 cell lysates. One possible explanation might be differences in expression levels of this protein among different cell lines coupled with the fact that Alix is enriched in exosome fractions; however, further in depth studies are necessary to completely explain this observation. Nonetheless, the presence of both exosomal markers and absence of calnexin verifies the purity of our exosome preparations.

We further confirmed this by analyzing exosomes using transmission electron microscopy (TEM). Representative TEM image of exosomes isolated from conditioned media of MDA-MB-231 cells as shown in Fig. 1 B demonstrated the presence of a homogeneous population of cup-shaped ∼100 nm exosome vesicles. Similar vesicles were also observed in preparations from conditioned media of both T47DA18 and MCF7 cells (data not shown). Taken together, these findings confirm the presence and purity of breast cancer cell secreted exosomes in our preparations.

To determine the exosome-HMEC interactions, we first assessed the uptake of breast cancer cell secreted exosomes by HMECs. To track exosome uptake, we labeled exosomes with a green fluorescent dye PKH-67 [52] and incubated sub-confluent layers of HMECs with PKH-67 labeled exosomes for up to 24 h. Uptake of exosomes by HMECs was assessed using fluorescence microscopy after extensively washing the cells to remove any extracellular exosomes. A representative image of HMECs incubated with exosomes from MDA-MB-231cells shown in Fig. 1 C demonstrates the uptake of exosomes by HMECs. Similar results were also observed for exosomes from T47DA18 and MCF7 cells (data not shown). In all cases we observed >90% of HMECs containing green fluorescent exosomes. We further confirmed this quantitatively, using flow cytometry to analyze uptake of PKH-67 labeled exosomes from MDA-MB-231 cells by HMECs (Fig. 1D). Although we did not study in depth the intracellular localization of these exosomes in the HMECs, preliminary data using LysoTracker to stain lysosomes [53] indicated some colocalization, suggesting that some of the exosomes were in lysosomal compartments of HMECs after their uptake (data not shown).

### Exosome-HMEC interactions induce ROS production in HMECs

Recently, the role of ROS induced autophagy in TME has been underscored by the proposal of an autophagic breast tumor stroma model that has been shown to play an important role in facilitating tumor growth and metastasis [54]. According to this new paradigm, cancer cells induce oxidative stress in cancer associated stromal cells [54]. Oxidative stress leads to induction of autophagy and senescence in stromal cells which results in the production of recycled nutrients and metabolites to “fuel” tumor growth [55]. This paradigm is supported by the following observations: (a) autophagy and senescence is noted in fibroblasts co-cultured with breast cancer cells [56]; (b) autophagic and senescent fibroblasts secrete L-lactate, ketone bodies and amino acids that serve as “fuels” for cancer cell growth [56]; (c) senescence is observed in cancer associated stromal cells in pathological human breast tumor sections [56], and (d) H_2_O_2_ induced senescent fibroblasts promote tumor growth and metastasis *in vivo* when co-injected with cancer cells in nude mice [57]. However, the precise nature of the signals coming from cancer cells that induces oxidative stress in stromal cells is not clearly understood.

**Figure 2:**
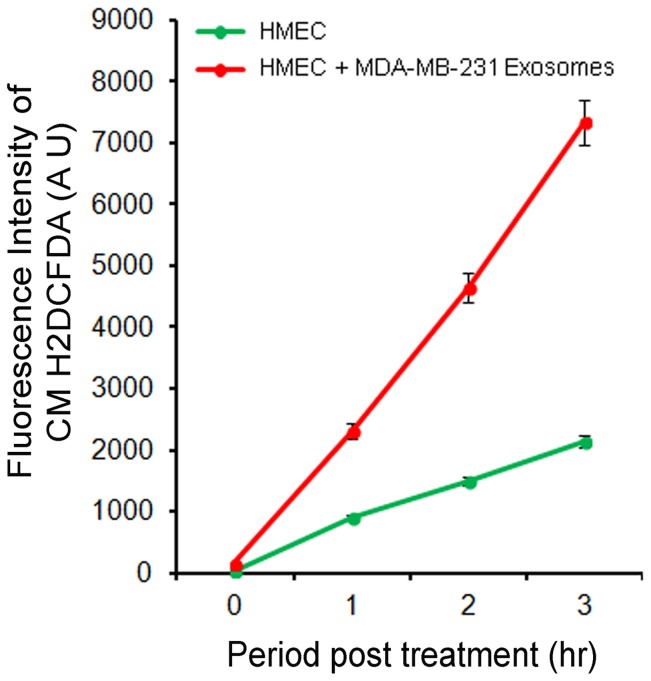
Detection of ROS production during exosome-HMEC interactions. Semi-confluent layers of 5×10^4^ HMECs were incubated with 10 µg protein equivalent of exosomes from MDA-MB-231 cells and ROS detection agent 10 µM CMH2DCFDA in a total volume of 300 µl of epithelial cell basal growth media for up to 3 h. Fluorescence of oxidized CMH2DCFDA was assessed fluorimetrically at the indicated time points to detect ROS production during exosome-HMEC interactions.

We investigated whether interactions and uptake of cancer cell released exosomes by HMECs serve as a signal to induce ROS in the mammary epithelial cells. We assessed the kinetics of ROS production in HMECs incubated with exosomes for up 3 h by fluorimetry using a cell permeable fluorogenic ROS probe CMH2DCFDA [58] (Fig. 2). Compared to the control HMECs alone, we detected significantly higher levels of ROS in HMECs incubated with exosomes from MDA-MB-231 cells (Fig. 2, red vs. green lines). Similar observations were noted when exosomes from T47DA18 and MCF7 cells were used (data not shown).

### Exosome-HMEC interactions induce autophagy in HMECs

**Figure 3:**
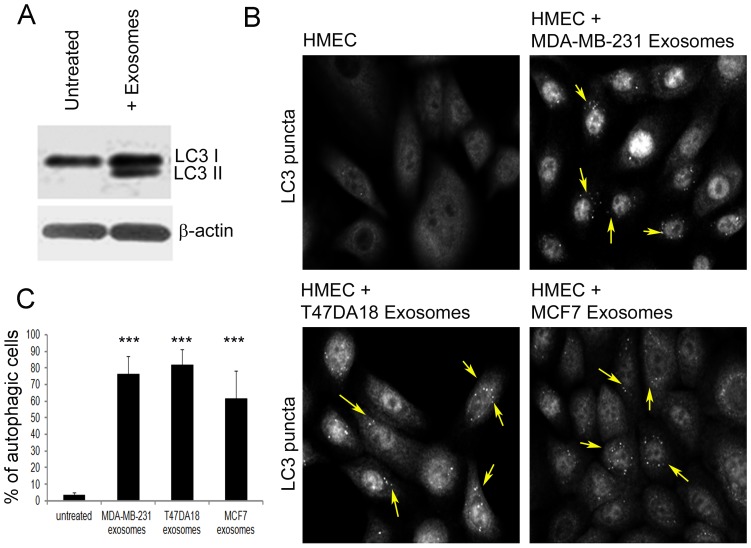
Induction of autophagy in HMECs following uptake of breast cancer cell released exosomes. (A) Western blot analysis for detection of proteins LC3 I and II in cellular lysates of untreated HMECs and those incubated with exosomes from MDA-MB-231 cells for 24 h. Equal protein concentrations of whole cell lysates were analyzed by western blots. β- actin was used as an equal loading control. (B) IFA of LC3 “puncta” formation in HMECs untreated or incubated with exosomes from either MDA-MB-231, T47DA18 or MCF7 cells for 24 h. Cells were washed, fixed with paraformaldehyde, permeabilized with saponin, blocked with normal donkey sera and reacted with rabbit polyclonal anti-LC3 antibodies. LC3 expression was detected using donkey anti-rabbit IgG secondary antibodies labeled with Alexa 594 fluorophore. White arrows indicate LC3 “puncta” characteristic of autophagy. (C) Quantitation of cells with LC3 puncta in cultures incubated with or without exosomes. A minimum of 10 independent fields of view/50 cells were chosen for colocalization analysis. Error bars indicate SEM values.***: *p*<0.001.

Next, we examined the induction of autophagy in HMECs following the uptake of exosomes. During autophagy, the microtubule-associated protein 1A/1B-light chain 3 (LC3; LC3 I) is cleaved and then conjugated to phosphatidylethanolamine to form LC3-phosphatidylethanolamine conjugate (LC3-II), which is then recruited to autophagosomal membranes [59]. To assess autophagy, we performed western blotting to detect the presence of autophagic proteins LC3 I and LC3 II [60], and IFA to detect cytoplasmic LC3 positive autophagosomal membranes or “LC3 puncta” [61] in HMECs incubated with exosomes for up to 24 h. While expression of only LC3 I was detectable in total cellular lysates of untreated HMECs, both LC3 I and II were clearly detected in lysates of HMECs incubated with exosomes from MDA-MB-231 cells for up to 24 h (Fig. 3 A). Similarly, using IFA, we did not detect any “LC3 puncta” in untreated HMECs and in contrast, numerous cytoplasmic “LC3 puncta” were observed in the HMECs exposed to exosomes from MDA-MB-231, T47DA18 or MCF7 cells, respectively (Fig. 3 B, yellow arrows). Quantitative assessment of “LC3 puncta” positive autophagic cells further showed that while these cells accounts for <5% of untreated HMECs, they are >60% of the population in the case of HMECs exposed to exosomes (Fig. 3 C). It is also interesting to note that we did not observe any significant difference in the number of autophagic cells when HMECs were incubated with exosomes from different types of breast cancer cells.

### Exosome-HMEC interaction induced ROS plays a role in autophagy induction in HMECs

**Figure 4:**
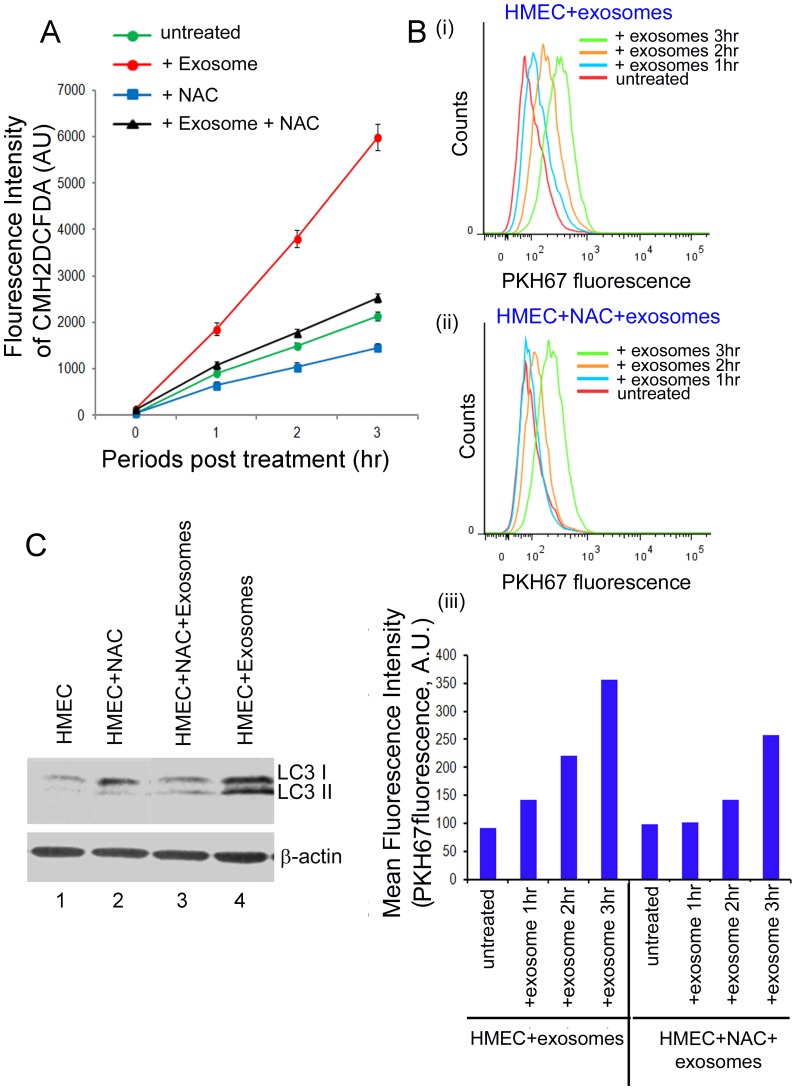
Effects of NAC on ROS production, exosome uptake and induction of autophagy during exosome-HMEC interactions. (A) HMECs were treated with or without NAC were incubated with or without exosomes from MDA-MB-231 cells for up to 3 h. ROS production was detected fluorimetrically using CMH2DCFDA at the indicated times. (B) Flow cytometry analysis of the effects of NAC on uptake of exosomes from MDA-MB-231 cells. HMECs were incubated with exosomes labeled with PKH-67 dye for different time periods and exosome uptake was assessed by flow cytometry (i). (ii) HMECs were treated with µM NAC for 1 hr and then incubated with PKH-67 labeled exosomes in the presence of NAC for different time periods and analyzed by flow cytometry. (iii) Comparisons of mean fluorescence intensities of HMECs under conditions described in (i) and (ii). (C) Western blot analysis for detection of autophagy protein LC3 I and II in cellular lysates of HMECs that were treated with or without NAC and incubated with or without exosomes from MDA-MB-231 cells for 3 h. Equal protein concentrations of cellular lysates were analyzed by western blots. β- actin was used as an equal loading control.

To determine whether the ROS induction during exosome-HMEC interactions serves as the “signal” for autophagy induction in HMECs, we used NAC (N-acetyl-L-cysteine), a scavenger of ROS [62], to inhibit ROS production in HMECs during exposure to cancer cell released exosomes. Subsequently, under optimum conditions of NAC treatment, we assessed for autophagy to determine if inhibition of ROS production during exosome-HMEC interactions led to inhibition of autophagy in HMECs. We used 1 µM NAC to inhibit ROS in HMECs as this was the highest concentration of NAC that did not show any cytotoxicity against HMECs when cells were treated for up to 4 h (data not shown). To measure ROS during NAC treatment, we pretreated HMECs with 1 µM NAC for 1 hr, washed and exposed them to exosomes for up to 3 h in the presence or absence of 1 µM NAC. 1 µM NAC treatment inhibited the naturally produced ROS in untreated HMECs (Fig. 4 A, untreated vs. NAC). To compare the kinetics of ROS production, HMECs were pre-treated with 1 µM NAC for 1 h and exposed to exosomes from MDA-MB-231 cells in the presence of 1 µM NAC for up to 3 h. NAC treatment not only significantly inhibited exosome induced ROS production for up to 3 h but also kept ROS levels comparable to background levels observed in untreated HMECs (Fig. 4 A, compare Exosome alone vs. Exosome +NAC vs. untreated).

Next, we assessed the effect of NAC treatment on exosome uptake during exosome-HMEC interactions. We used 1 µM NAC under conditions as described above for ROS assessments during exosome-HMEC interactions. HMECs were incubated with PKH-67 dye exosomes derived from MDA-MB-231 cells, in the presence or absence of NAC for up to 3 h. At different time intervals HMECs were washed and analyzed by flow cytometry to assess uptake of PKH-67 dye labeled exosomes (Fig. 4 B). A progressive increase in the population of HMECs containing PKH-67 positive exosomes was observed in HMECs incubated with exosomes in the absence of NAC (Fig. 4 B, i), indicating an increase in exosome uptake over time. However, in the presence of NAC, the uptake of exosomes by HMECs was observed to be reduced but not completely abrogated (Fig. 4 B, ii and iii). These findings indicate that while NAC treatment does not completely abrogate exosome uptake by HMECs, it results in significant inhibition of ROS production in HMECs.

Finally, we assessed LC3 by western blotting to determine if ROS inhibition by NAC under optimal conditions as described in Fig. 4 A (i.e., HMEC+MDA-MB-231 exosomes +1 µM NAC for 3 h) inhibits autophagy in HMECs. β- actin levels were used as loading control. We detected only LC3 I in untreated HMECs and in HMECs treated with NAC for 3 h (Fig. 4 C, lanes 1 and 2). However, while both LC3 I and II were observed in HMECs exposed to exosomes for 3 h in the absence or presence of NAC, LC3 II levels were significantly decreased in the presence of NAC (Fig. 4 C, lanes 3 vs. 4). Taken together these findings suggested that interaction of HMECs with exosomes from breast cancer cells induce ROS, which can further result in autophagy induction in these epithelial cells.

### ROS produced during exosome-HMEC interactions results in induction of DNA damage response (DDR)

**Figure 5:**
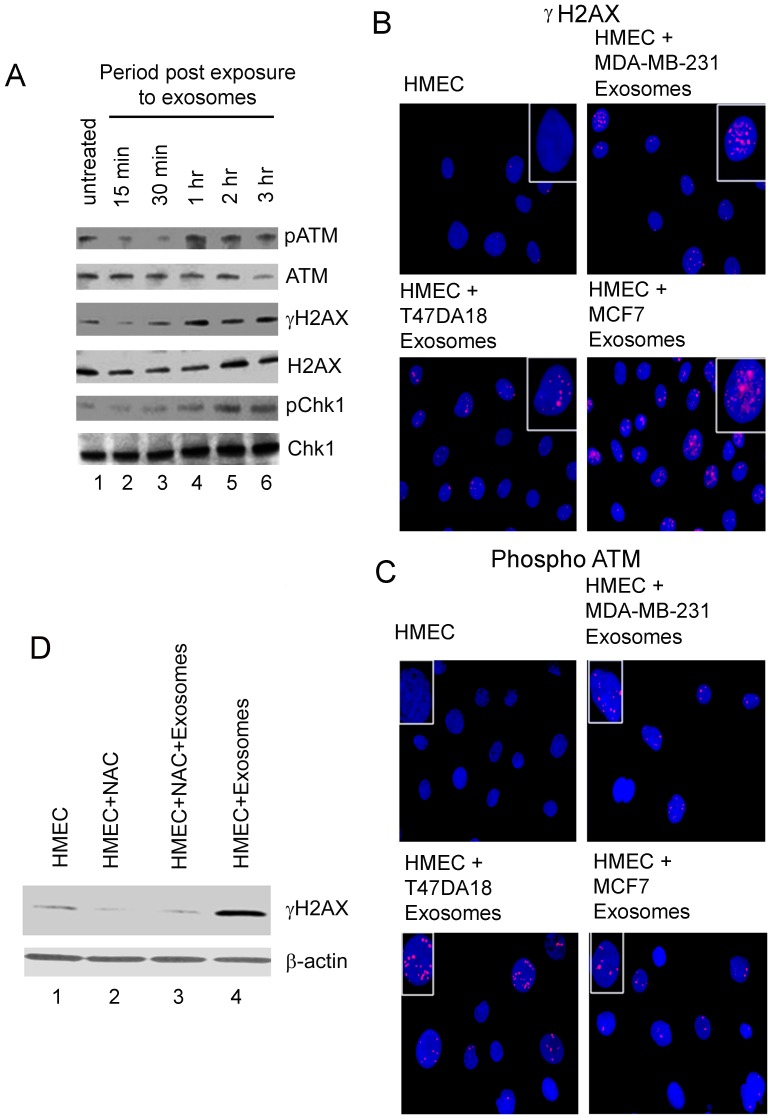
Detection of DNA damage response in HMECs incubated with exosomes and its abrogation by NAC. (A) Western blot analysis for expression of phosphorylated ATM (pATM), H2AX (γH2AX), and Chk1 (pChk1) in untreated HMECs and those incubated with exosomes from MDA-MB-231 cells for up to 3 h. Equal protein concentrations of cellular lysates were analyzed by western blots for phosphorylated and total protein levels. (B) IFA of phosphorylated H2AX (γH2AX) “micronuclei” formation in HMECs untreated or incubated with exosomes from either MDA-MB-231, T47DA18 and MCF7 cells for 24 h. Cells were washed, fixed with paraformaldehyde, permeabilized with saponin, blocked with normal donkey sera and reacted with mouse polyclonal anti-phospho H2AX antibodies. γH2AX expression was detected using donkey anti-rabbit IgG secondary antibodies labeled with Alexa 594 fluorophore. Nuclei were stained with DAPI. (C) IFA of phospho ATM in HMECs untreated or incubated with exosomes from either MDA-MB-231, T47DA18 and MCF7 cells for 24 h. Cells processed as described in (B) and reacted with rabbit polyclonal anti-phospho ATM antibodies. phospho ATM expression was detected using donkey anti-rabbit IgG secondary antibodies labeled with Alexa 594 fluorophore. Nuclei were stained with DAPI. (D) Western blot analysis for expression of phosphorylated H2AX (γH2AX) in HMECs that were untreated, treated with NAC alone, treated with NAC and incubated with exosomes, or left untreated but incubated with exosomes from MDA-MB-231 cells for 3 h. Equal protein concentrations of cellular lysates were analyzed. β- actin was used as an equal loading control.

ROS induced oxidative stress is known to induce DDR [63] in cells which can lead to both phosphorylation of p53 at serine 15, resulting in stabilization of p53 [64]. Moreover, stabilization of p53 can lead to either apoptosis or autophagy and senescence via cell cycle arrest [65], [66]. To determine the possible mechanism by which ROS, produced during exosome-HMEC interactions can induce autophagy, we assessed the induction of DDR. HMECs incubated with exosomes from MDA-MB-231 cells for up to 3vh were analyzed by western blotting to detect the phosphorylation of ATM, H2AX (γH2AX), and Chk1, 3 key members of the DDR pathway [67] (Fig. 5 A). Compared to the levels in untreated HMECs, levels of phospho ATM, H2AX and Chk1 increased significantly with the increase in time of incubation of HMECs with exosomes (Fig. 5 A). Total levels of ATM, H2AX, and Chk1 did not show any significant change during this period. These findings clearly indicated that exosome-HMEC interactions lead to DDR induction.

To further assess whether DDR is induced in HMECs by exosomes from all 3 breast cancer cells, we performed IFA to detect γH2AX specific micronuclei formation and phospho ATM in HMECs incubated with exosomes for up to 24 h (Fig. 5, B and C). Representative images of IFA for γH2AX and phospho ATM in the nuclei of untreated HMECs vs. HMECs incubated with exosomes as shown in Fig. 5 B and C, clearly demonstrated that γH2AX micronuclei and phospho ATM were only detectable in HMECs incubated with exosomes. To further determine whether ROS induced during exosome-HMEC interactions was the signal for induction of DDR, we performed western blots for γH2AX in untreated HMECs and those treated with or without 1 µM NAC and incubated with exosomes from MDA-MB-231 cells for 3 h (Fig. 5 D). We observed that compared to basal levels of γH2AX in untreated HMECs, NAC treatment alone did not increase levels of γH2AX (Fig. 5 D, lanes 1 vs. 2). Moreover, while we detected significantly higher levels of γH2AX in HMECs incubated with exosomes, only low levels of γH2AX (comparable to basal levels observed in untreated HMECs) were detected in HMECs treated with NAC and incubated with exosomes (Fig. 5 D, compare lanes 1, 3 and 4). These findings suggest that inhibition of ROS produced during exosome HMEC interaction, by NAC inhibits DDR in HMECs.

### ROS produced during exosome-HMEC interactions results in p53 stabilization

**Figure 6:**
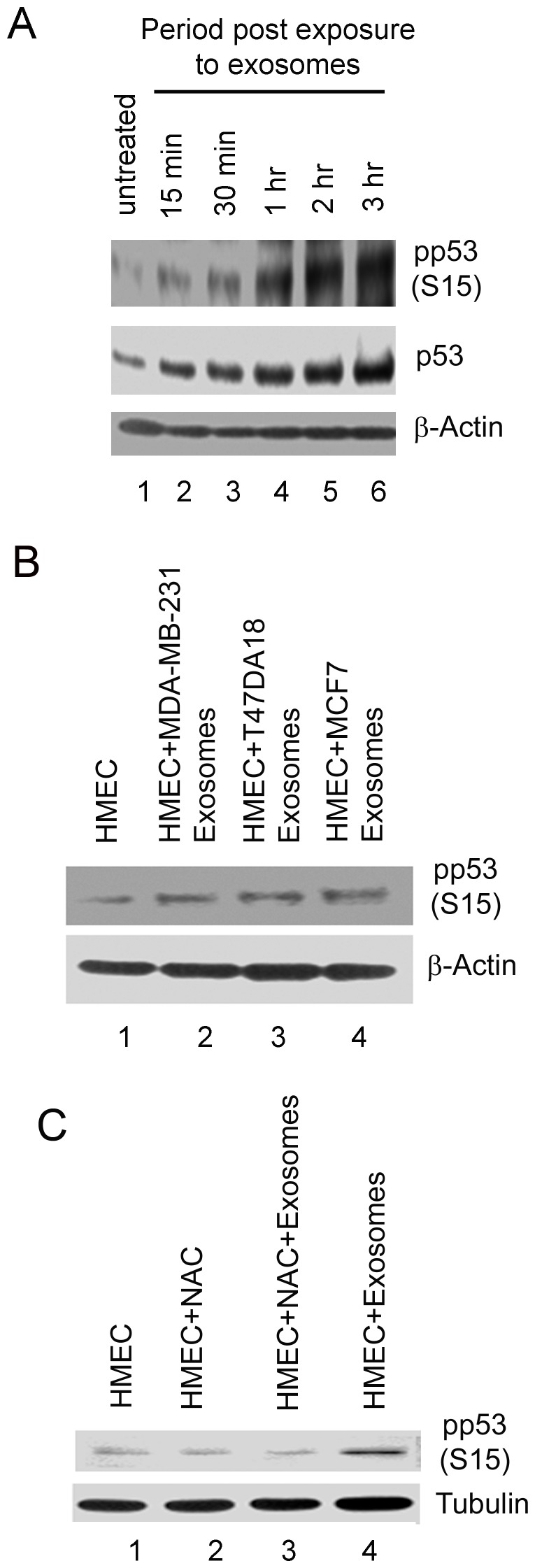
Detection of phosphorylation of p53 in HMECs incubated with exosomes and its abrogation by NAC. (A) Western blotting for detection of phosphorylation of p53 at serine 15 (pp53 S15) in HMECs incubated with exosomes from MDA-MB-231 cells for up to 3 h. Equal protein concentrations of cellular lysates were analyzed by western blots for pp53 S15 and total levels of p53. β- actin was used as an equal loading control. (B) Western blot analysis for pp53 S15 in cellular lysates of HMECs untreated and those treated with exosomes from 3 different breast cancer cell lines, MDA-MB-231, T47DA18 and MCF7 respectively for 24 h. β- actin was used as an equal loading control. (C) Western blot analysis for pp53 S15 in cellular lysates of HMECs untreated, treated with NAC alone, treated with NAC and incubated with exosomes, in untreated but incubated with exosomes from MDA-MB-231 cells for 3 h. Tubulin was used as an equal loading control.

Phosphorylation of p53 at S15 is well known to stabilize p53 by preventing its proteosomal degradation [68]. To investigate whether p53 is stabilized during exosome- HMEC interactions, we performed western blots to detect the phosphorylation of p53 at the serine 15 residue (pp53 S15) in HMECs incubated with exosomes from MDA-MB-231 cells for up to 3 h (Fig. 6 A, top panel). β-actin levels were used as a loading control. We observed a progressive increase in phosphorylation at S15 during the entire period of exosome-HMEC interaction (Fig. 6 A, top panel). Furthermore, we also observed that total p53 levels significantly increased over time (Fig. 6 A, middle panel) indicating that S15 phosphorylation leads to stabilization of p53 under the above conditions. We also checked the phosphorylation state of the S9, S46 and S392 residues of p53 [69], and did not observe any change indicating that these sites were not affected (data not shown). Furthermore, we observed that S15 phosphorylation of p53 (pp53 S15) was sustained until 24 hours post incubation of HMECs with exosomes from all three breast cancer cell lines, MDA-MB-231, 47DA18 and MCF7 cells, respectively (Fig. 6 B, lanes 2 to 4 vs. lane 1). Lastly, we compared pp53 S15 levels in untreated HMECs vs. those in HMECs treated with or without 1 µM NAC and incubated with exosomes from MDA-MB-231 cells for 3 h (Fig. 6 C). We observed almost complete prevention of S15 phosphorylation of p53 (pp53 S15) in HMECs incubated with exosomes in the presence of NAC in comparison to levels of pp53 S15 noticed in the absence of NAC and in untreated controls (Fig 6 C, lanes 3 vs. 1, 2 and 4). Taken together, our data so far have indicated that exosome induced ROS results in induction of the DDR and stabilization of p53 via phosphorylation at S15 during exosome-HMEC interactions.

### Exosome-HMEC interactions result in secretion of breast cancer cell growth promoting factors

**Figure 7:**
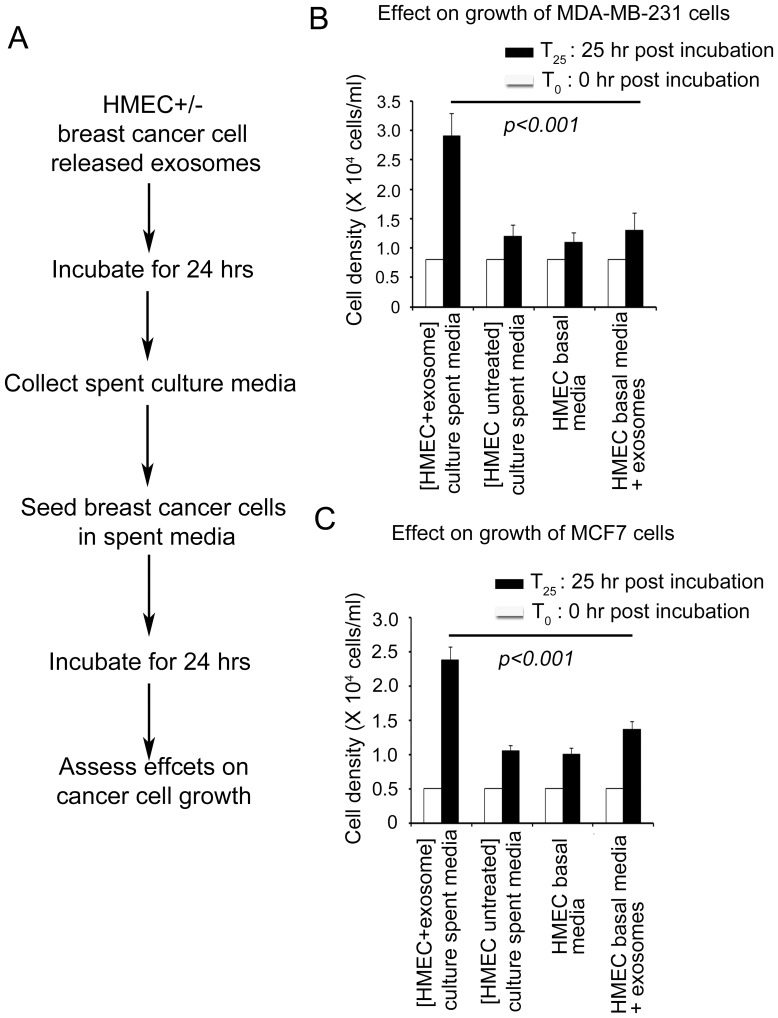
Effects of conditioned media from HMECs incubated with exosomes on growth of breast cancer cells. (A) Schematics of experimental design. HMECs were untreated or incubated with exosomes from MDA-MB-231 and MCF7 cells respectively in human epithelial cell basal culture media for 24 h. Spent media from HMEC cultures exposed to exosomes was collected and filtered using a 0.22 µm sterile filter and used as culture media to grow breast cancer cell lines for 24 h as described in materials and methods. (B) Growth of MDA-MB-231 cells in spent media from HMECs incubated with exosomes from MDA-MB-231 cells and controls, spent culture media from untreated HMECs, HMEC basal growth media and HMEC basal growth media supplemented with exosomes from MDA-MB-231 cells. (C) Growth of MCF7 cells in spent culture media from HMECs incubated with exosomes from MCF7 cells and controls, spent culture media from untreated HMECs, HMEC basal growth media and HMEC basal growth media supplemented with exosomes from MCF7 cells.

Several recent studies have shown that autophagic breast cancer associated fibroblasts can promote tumorigenesis and metastasis of breast cancer cells by releasing growth promoting “metabolites” and amino acids [70]. We investigated whether autophagy induced in HMECs by breast cancer cell released exosomes could also facilitate cancer cell growth. In separated experiments we exposed HMECs to exosomes from either MDA-MB-231 or MCF7 cells, in HMEC basal media for up to 24 h (optimal conditions that have been observed to induce autophagy in HMECs as shown in Fig. 3). Spent media from HMEC cultures exposed to exosomes were passed through a 0.22 µm sterile filter and tested for its ability to promote growth of the same breast cancer cells (Fig. 7 A). Growth of breast cancer cells (i.e., MDA-MB-231 and MCF7, respectively, Fig. 7 B and C, respectively) in spent media from HMEC cultures exposed to exosomes was compared to controls such as (a) conditioned media from exosome untreated HMECs, (b) HMEC basal culture media, and (c) HMEC basal media containing exosomes. We observed that while all control media (as described above) supported growth of cancer cells to a similar extent (up to 2.25 fold increase), only spent media from HMEC cultures exposed to exosomes promoted a significant increase in cancer cell growth by up to ∼4–5 fold (Fig. 7 B and C).

## Discussion

**Figure 8:**
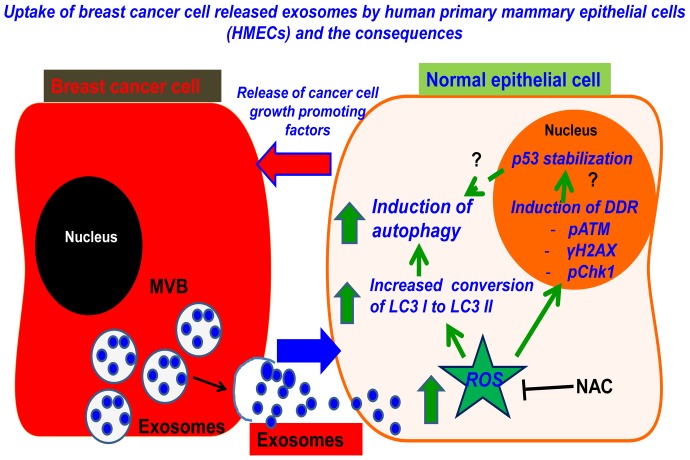
Proposed model for breast cancer cell and HMEC crosstalk. Exosomes released from breast cancer cells interact and are taken up by HMECs. Exosome-HMEC interactions induce ROS, which further induces autophagy, phosphorylation of ATM, H2AX and Chk1 (DDR) and stabilization of p53. Inhibition of ROS by NAC abrogates autophagy, DDR and stabilization of p53. Exosome induced autophagic HMECs release breast cancer cell growth promoting factors.

The findings of our study show that breast cancer cell released exosomes can induce autophagy, DDR and p53 stabilization via ROS production, in HMECs and the autophagic HMECs release breast cancer cell growth promoting factors (Fig. 8). To the best of our knowledge, this is the first report to indicate that ROS generated during exosome-target cell interactions may be a possible mechanism by which autophagy can be induced in target cells but also underscores the role of autophagic HMECs in promoting tumorigenesis.

In this study we provide evidence that breast cancer cell released exosomes are taken up by HMECs and furthermore report the biological functions mediated by the exosomes. While cancer cell secreted exosomes are largely regarded as a treasure trove for biomarkers [25], [27], the biological functions mediated by these exosomes may represent one of the most intriguing mechanisms by which cancer cells manipulate the tumor microenvironment to create a “niche” for tumorigenesis [71]. Biological functions carried out by breast cancer cell secreted exosomes are relatively unknown in comparison to those in other cancer types. Here we studied some of the biological functions mediated by exosomes secreted by 3 different breast cancer cell lines, MDA-MB-231, T47DA18 and MCF7, representing 3 different types of breast cancers [48]–[50]. Interestingly, we observed that all 3 breast cancer cell lines secreted similar amounts of exosomes. However, further clinical studies are necessary to ascertain whether different types and stages of breast cancers secrete similar or different amounts of exosomes and also if there is heterogeneity among the exosomes secreted. Nonetheless, while we did not study the precise mechanism of exosome-HMEC interaction, our studies show that exosomes from different breast cancer cell lines are similarly taken up by HMECs and produced similar phenotypes (e.g. ROS production, autophagy, DDR and p53 stabilzation) in them. However, since exosomes are believed to bear molecular signatures of cells they are secreted from, diversity with respect to the nature of the exosomal cargo in exosomes originating from different types of breast cancer cells can be easily envisioned, this is also predicted to contribute to manifestation of phenotypic differences in HMECs other than those observed by us. Furthermore, while in this study we have focused on HMECs, given the complexity and heterogeneity in the composition of the TME, interactions between cancer cell released exosomes and other cells of TME also needs to addressed. Nonetheless, to the best of our knowledge, this study represents the first report of biological consequences of interactions between breast cancer exosomes and primary HMECs.

Some key findings of our studies (Fig. 8) here include the observed ROS production during exosome HMEC interactions and its role in induction of autophagy in HMECs. The role of autophagy in tumorigenesis has been extensively studied by many groups [54]–[57]. It is perhaps best described as compartment and cell type specific, particularly due to observations such as the “Autophagy Paradox” [55]. While several reports have indicated that autophagy in cancer cells effectively suppress tumorigenesis, recent studies have indicated that autophagy in the TME may promote tumor growth via supply of nutrients and “reverse Warburg effect” [55], [70]. Interestingly, these studies using co-culture systems of breast cancer cell lines and fibroblasts have shown that ROS are generated and induces autophagy in tumor associated fibroblasts. Furthermore, ROS producer H_2_O_2_, has been shown to induce autophagy and senescence in TME [57]. While the source of ROS in theTME remains unclear, the observed phenomena is described as the autophagy – senescence transition in TME and has been proposed to explain the link between breast cancer onset and aging [57]. Interestingly, while our studies are in line with others [55], [57] and demonstrate that breast cancer cells are responsible for induction of ROS that induce autophagy in HMECs, we here identify breast cancer cell secreted exosomes as the inducer of ROS in these epithelial cells. Furthermore using NAC to inhibit exosome induced ROS we demonstrate abrogation of ROS induced autophagy. However, additional studies are necessary to delineate the precise mechanism behind ROS production during exosome-HMEC interactions.

In attempt to study the possible mechanism by which exosome induced ROS in turn induces autophagy, we assessed the involvement of DDR and p53. ROS is a well characterized inducer of DNA damage and activation of p53 [63], [64]. ROS mediated DNA damage are known to engage double-stranded DNA repair mechanisms (DDR) [67]. These mechanisms include initiation of a signaling cascade involving ATM/ATR, the local deposition of 53BP1/γH2AX (micronuclei foci formation) and modulation of cell cycle regulation by Chk1/2 [67], [72]. ATM is activated via double stranded breaks while ATR responds to single strand damage [67]. ATM/ATR has been shown to phosphorylate p53 at serine 15, which eventually leads to the stabilization and activation of p53 [69]. Continuous activation of p53 leads to induction of autophagy, senescence or apoptosis [73]. We not only observed phosphorylation of H2AX, ATM, Chk1 and p53 at S15 and its stabilization during exosome-HMEC interactions, but interestingly, we also observed that DDR was induced as early as 1 h post incubation of HMECs with exosomes, indicating that ROS production and not uptake of exosomes may be the major signal for this process, since 1 h incubation resulted in only <20% of HMECs containing exosomes. Furthermore, we also demonstrated that abrogation of ROS production during exosome-HMEC interactions by NAC prevented phosphorylation of H2AX and p53. While these observations suggest that these mechanisms may contribute to induction of autophagy, further studies are necessary to establish whether DDR and p53 phosphorylation are linked or mutually independent events induced by ROS.

Finally, we demonstrate that only conditioned media from exosome treated HMECs can promote cancer cell growth. Our data clearly indicates that exosomes themselves do not serve as carriers of growth factors for cancer cells since HMEC basal media supplemented with exosomes do not significantly promote cancer cell growth when compared with HMEC basal media alone or conditioned media from HMECs not exposed to cancer cell exosomes. These findings clearly indicate that autophagic HMECs produced by exosome-HMEC interactions secrete cancer cell growth promoting factors. While we did not study whether a “reverse Warburg effect” and nutrient recycling are possible mechanisms involved here, our observations of promoting cancer cell growth by conditioned media from autophagic HMECs are in agreement with those reported by others using *in vitro* co-culture systems or co-inoculation in animal models of autophagic fibroblasts and breast cancer cells [70].

## Conclusions

Our studies here not only underscores the functional role of breast cancer secreted exosomes in manipulating the tumor microenvironment to promote cancer cell growth but also establishes the role of normal mammary epithelial cells in tumorigenesis. The significance of exosome mediated manipulation of these epithelial cells are underscored by the fact that not only do these cells make up the mammary ductal microenvironment of the terminal ductal lobular unit which is the origin of most pathologic breast lesions [74], but also because these cells are found in the TME of invasive breast tumors [5], [7]. Further studies to delineate the mechanism of exosome -HMEC interactions and characterization of the exosome induced secretome of these cells are expected to lead to the development of new avenues for prevention and intervention of breast cancer.

## Supporting Information

Figure S1 **Schematics of method of exosome isolation from cell conditioned media of breast cancer cells.**
(TIF)
