# Supplementary figures and images for: Interactions between Exosomes from Breast Cancer Cells and Primary Mammary Epithelial Cells Leads to Generation of Reactive Oxygen Species Which Induce DNA Damage Response, Stabilization of p53 and Autophagy in Epithelial Cells
Source: PLoS One. 2014 May 15;9(5):e97580. doi: 10.1371/journal.pone.0097580 (PMC4022578; doi:10.1371/journal.pone.0097580)

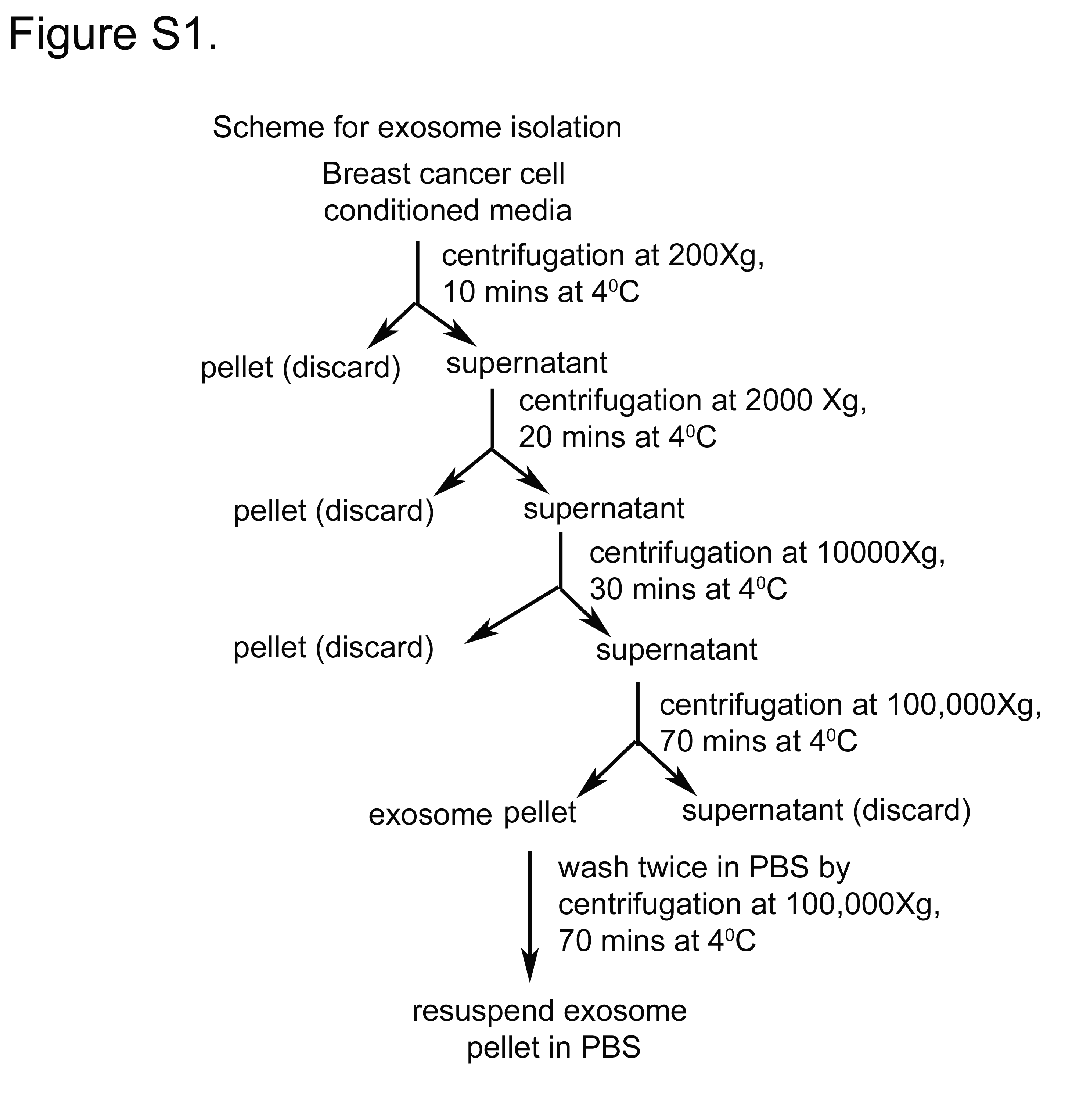

Supplement: Figure S1 — Schematics of method of exosome isolation from cell conditioned media of breast cancer cells. (TIF) [file pone.0097580.s001.tif]
